# Efficient and Robust NK-Cell Transduction With Baboon Envelope Pseudotyped Lentivector

**DOI:** 10.3389/fimmu.2019.02873

**Published:** 2019-12-16

**Authors:** Aurelien B. L. Colamartino, William Lemieux, Panojot Bifsha, Simon Nicoletti, Nitin Chakravarti, Joaquín Sanz, Hugo Roméro, Silvia Selleri, Kathie Béland, Mélanie Guiot, Camille Tremblay-Laganière, Renée Dicaire, Luis Barreiro, Dean A. Lee, Els Verhoeyen, Elie Haddad

**Affiliations:** ^1^Department of Microbiology, Infectiology and Immunology, University of Montréal, Montréal, QC, Canada; ^2^CHU Sainte-Justine Research Center, Montréal, QC, Canada; ^3^INSERM U1163 and CNRS ERL 8254, Medicine Faculty, Paris Descartes University, Necker Hospital, Paris, France; ^4^Department of Medical Oncology, Thomas Jefferson University, Philadelphia, PA, United States; ^5^Institute for Bio-computation and Physics of Complex Systems (BIFI), University of Zaragoza, Zaragoza, Spain; ^6^Department of Theoretical Physics, Faculty of Sciences, University of Zaragoza, Zaragoza, Spain; ^7^Pierre and Marie Curie University (PMCU) Paris 6, Paris, France; ^8^Assistance Publique Hopitaux De Paris (AP-HP), Paris, France; ^9^Genetics Section, Department of Medicine, University of Chicago, Chicago, IL, United States; ^10^Center for Childhood Cancer and Blood Disorders, Research Institute of Nationwide Children's Hospital, Columbus, OH, United States; ^11^CIRI, Université de Lyon, INSERM U1111, ENS de Lyon, Université Lyon 1, CNRS UMR 5308, Lyon, France; ^12^Université Côte d'Azur, INSERM, C3M, Nice, France; ^13^Department of Pediatrics, University of Montréal, Montréal, QC, Canada

**Keywords:** NK-cell transduction, baboon retrovirus envelope pseudotyped lentivectors, chimeric antigen receptor, NK amplification and expansion system (NKAES), cytotoxicity

## Abstract

NK-cell resistance to transduction is a major technical hurdle for developing NK-cell immunotherapy. By using Baboon envelope pseudotyped lentiviral vectors (BaEV-LVs) encoding eGFP, we obtained a transduction rate of 23.0 ± 6.6% (mean ± SD) in freshly-isolated human NK-cells (FI-NK) and 83.4 ± 10.1% (mean ± SD) in NK-cells obtained from the NK-cell Activation and Expansion System (NKAES), with a sustained transgene expression for at least 21 days. BaEV-LVs outperformed Vesicular Stomatitis Virus type-G (VSV-G)-, RD114- and Measles Virus (MV)- pseudotyped LVs (*p* < 0.0001). mRNA expression of both BaEV receptors, ASCT1 and ASCT2, was detected in FI-NK and NKAES, with higher expression in NKAES. Transduction with BaEV-LVs encoding for CAR-CD22 resulted in robust CAR-expression on 38.3 ± 23.8% (mean ± SD) of NKAES cells, leading to specific killing of NK-resistant pre-B-ALL-RS4;11 cell line. Using a larger vector encoding a dual CD19/CD22-CAR, we were able to transduce and re-expand dual-CAR-expressing NKAES, even with lower viral titer. These dual-CAR-NK efficiently killed both CD19^KO^- and CD22^KO^-RS4;11 cells. Our results suggest that BaEV-LVs may efficiently enable NK-cell biological studies and translation of NK-cell-based immunotherapy to the clinic.

## Introduction

The relative resistance of NK cells to transduction hampers the study of NK-cell biology and the development of NK cell-based immunotherapy. VSV-G-LVs, classically used to generate chimeric antigen receptor (CAR)-T cells (1), do not efficiently transduce NK-cells. RD114-pseudotype viral vectors represent an attractive alternative since their entry receptor, the sodium-dependent neutral amino acid transporter (ASCT2) (2, 3), is widely expressed in the hematopoietic lineage (4). However, despite encouraging initial report, RD114-based viral vectors only transduce NK-cells at low levels. Nevertheless, they recently enabled clinical development of cord-blood derived CAR-NK-cells (5). To fill the need for an efficient method for transducing NK cells, we investigated alternative pseudotyping proteins. Since the Baboon envelope pseudotyped lentiviral vector (BaEV-LV) binds ASCT1 (6) in addition to ASCT2 for viral entry, we assessed their efficacy to transduce NK-cells for therapeutic purposes. As observed independently by Bari et al. (7) our data demonstrate the efficacy of BaEV-LV in NK-cell transduction.

## Methods

### Cells and Culture Condition

Blood samples were obtained from healthy volunteers after informed consent (IRB-approved protocol #CER-3527). NK-cells were enriched from PBMC using a CD56-positive selection kit (Stemcell Technologies, Canada). NK cells were expanded using the Amplification and Expansion System (NKAES) with irradiated K562mbIL21 or K562mbIL15 feeder cells as described (8, 9). Alternatively, NK-cells were amplified using NK-MACS Medium (130-114-429, Miltenyi) system according to manufacturer's instructions. RS4;11 (ATCC) CD19/22^KO^ were generated using purified Cas9 protein and two gRNA targeting CD19 or CD22 (Integrated DNA Technologies). CD19^KO^ and/or CD22^KO^ cells were FACS-sorted based on loss of surface marker expression. Cells were cultured in DMEM (Wisent) or RPMI1640 supplemented with 10% FCS and penicillin/streptomycin (Gibco). Media were supplemented with 100 UI/mL IL-2 (Proleukin—Novartis Pharmaceuticals, Canada) for NK-cell cultures.

### Plasmids and Viral Production

An UCOE sequence (10) was added to the lentiviral vector pHRSIN-SFFV-eGFP (11) upstream of the SFFV promoter to produce pHUS-GFP vector. For the CAR-expression vector, GFP in pHUS-GFP was replaced by an anti-CD22 CAR (m971 ScFv) fused to 28BBz constructed from 28z and BBz (Dr. Orentas, National Cancer Institute) (12). For the dual CAR-expression vector, the GFP-cassette was replaced by 2nd-generation anti-CD19 and anti-CD22 CARs, separated by a self-cleaving T2A peptide (**Figure 3A**). The pMD2.G (VSV-G) was a gift from Didier Trono (Addgene plasmid#12259; http://n2t.net/addgene:12259; RRID:Addgene_12259) and pLTR-RD114A (13) (RD114) was a gift from Jakob Reiser (Addgene plasmid#17576; http://n2t.net/addgene:17576; RRID:Addgene_17576). The Measles virus (MV-LV) and BaEVRLess envelope plasmids were used as previously described (6). Titration was performed on HEK293T cells (ATCC) using serial virus dilutions (6).

### Viral Transduction

NKAES were transduced after 1 week of expansion. One day before transduction, a 12-well plate was coated with RetroNectin (Takara). The following day, concentrated vectors at indicated multiplicity of infection (MOI), were added to coated plates for 4 h at 37°C. Then NK cells were seeded in these wells in IL-2-supplemented medium and protamine sulfate (Pharmaceutical Partners of Canada Inc.) (8 ug/mL). The plates were then centrifuged at 1,000 g for 1 h and incubated at 37°C overnight. The next day, IL-2-supplemented medium was added to each well. Transduction was assessed on day 3 or day 5 after transduction for NKAES and freshly isolated NK-cells (FI-NK), respectively.

### Flow Cytometry

All samples were stained with anti-CD56-APC, anti-CD3-FITC (Biolegend) and 7AAD (BD Biosciences). Transgene expression was detected by flow cytometry on 7AAD^−^ CD56(-APC)^+^ CD3(-PE)^−^ cells (Biolegend). For NK-cell receptor detection, samples were stained with DAPI, CD56-BV711, CD16-BV786, NKp30-AF647, NKp44-PE, NKp46-BV421 (Biolegend), NKG2D-APC (BD Biosciences), and NKG2A-PE (Miltenyi Biotec). CD3-BV650 and CD19-APC-Cy7 (Biolegend) markers were used as a gating exclusion strategy for the NK cell staining. Receptor expression was assessed on DAPI^−^ CD56(-BV711)^+^ CD3(-BV650)^−^ cells. To detect CAR-expression, cells were incubated with 2 μl Siglec2(CD22)-Fc chimera (50 mg/ml, R&D) for 30 min at 4°C, washed and stained with anti-Fc-PE (Jackson Immune).

### Cytotoxicity Assay

Cytotoxicity was assessed 24 h after cell contact by flow cytometry. Targets cells were loaded with PKH26 dye (Sigma-Aldrich) according to the manufacturer's directives and seeded in 96 well round bottom plates. Effector cells were then added at different effector:target ratios and medium alone was added to control wells. Before acquisition, 7AAD was added to each well to discriminate dead cells. The cytotoxicity was calculated as: Cytotoxicity (%) = [1-live targets (sample)/live targets (control)] × 100%.

### mRNA Quantification

RNA-seq expression studies were independently performed in two laboratories (Accession #GSE128696, #GSE129044). For the FI-NK vs. IL-21-NKAES/IL-15-NKAES comparisons, extraction of total RNA was done using the RNeasy mini kit (Qiagen) and Total RNA Purification Plus Kit (Norgen Biotek), respectively. The quality of RNA was verified with 2100 Bioanalyzer (Agilent) prior to preparation of sequencing libraries with the TruSeq RNA Sample Prep v2 Kit. Quality of libraries was verified via Agilent 4200 Tapestation using a High Sensitivity D1000 ScreenTape Assay kit. For the IL-15 NKAES analysis, approximately 60–80 million paired-end 150 bp sequence reads per library were generated, whereas for the IL-21 NKAES analysis, 30 million single-end 101 bp sequence reads per library were generated, both using Illumina HiSeq4000 platform. Kallisto, an RNA quantification program based on pseudoalignment was used to obtain read count estimates per gene (14). The differential gene expression analysis was done using DESeq2, edgeR, and limma R packages.

### Statistical Analyses

Statistical analyses were performed using GraphPad PRISM 8.0 (GraphPad Software). Statistical significance was determined by one-way or 2-way ANOVA with multiple testing and Bonferoni correction or using simple multiple *T*-tests with Holm-Sidak correction.

### Study Approval

Blood samples were obtained from healthy volunteers after informed and written consent. The study was approved by the institutional ethical board of the CHU Sainte-Justine (approved protocol #CER-3527).

## Results and Discussion

**Figure 1:**
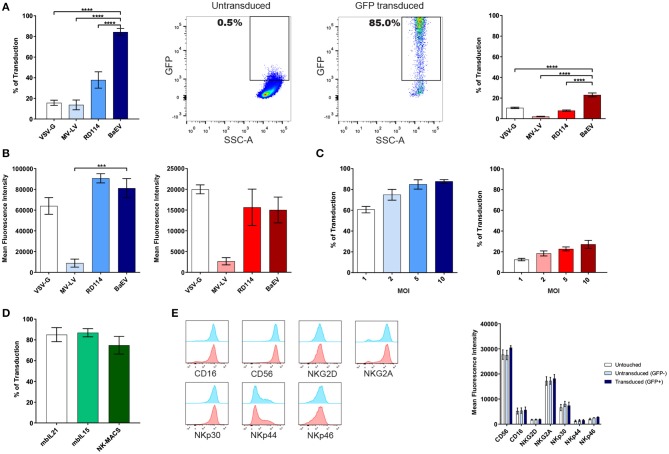
BaEV-LV efficiently transduces expanded (NKAES) and freshly isolated NK-cells (FI-NK). **(A)** Transduction of NK amplification and Expansion System (NKAES) cells (K562mbIL21 system, left panel-in blue) or FI-NK (right panel- in red) using VSV-G (*n* = 3 and *n* = 6), Measles virus (MV; *n* = 3 each), RD114 (*n* = 7 and *n* = 6) or BaEV (*n* = 8 and *n* = 12) envelope pseudotyped LVs encoding eGFP at a MOI of 10. Fluorescence was evaluated by flow cytometry (****P < 0.0001 for both NKAES and FI-NK; one-way ANOVA test with multiple testing and Bonferroni correction). On the middle panels are shown representative flow cytometry plots of GFP expression after NKAES-cell transduction with BaEV-LVs (untransduced, middle left; transduced, middle right). **(B)** Mean fluorescence intensity for GFP expression in NKAES cells (K562mbIL21 system, left panel-in blue) or FI-NK (right panel-in red) using VSV-G (*n* = 3 and *n* = 6), Measles virus (MV; *n* = 3 each), RD114 (*n* = 3 and *n* = 6) or BaEV (*n* = 8 and *n* = 12) envelope pseudotyped LVs encoding eGFP. Fluorescence was evaluated by flow cytometry (***p < 0.001 and *p* = 0.0882 for NKAES and FI-NK respectively; one-way ANOVA test with multiple testing and Bonferroni correction). **(C)** Transduction of NKAES (left panel-blue; *n* = 5) or FI-NK (right panel-red; *n* = 3) using BaEV-LV-encoding eGFP at various multiplicities of infection (MOI) was measured by fluorescence. **(D)** BaEV-LV transduction of NKAES using K562mbIL21 or K562mbIL15 irradiated feeder cells (*n* = 4), or using the NK-MACS Medium (*p* = 0.4265; one-way ANOVA test with multiple testing and Bonferroni correction, *n* = 3). **(E)** Left panel: Flow cytometry plot representative of NK cell markers expression after NK-cell transduction with BaEV-LVs. Transduced (GFP positive) NKAES are in blue and non-transduced (GFP negative) NKAES are in red. Right panel: Mean fluorescence intensity for NK cell markers for untouched and BaEV-LV treated NKAES. BaEV-LV treated NKAES were gated according to GFP to separate transduced (GFP positive) or non-transduced (GFP negative) NKAES (*p* = 0.2994; 2-way ANOVA test with multiple testing and Bonferroni correction, *n* = 4). Data are presented as the mean ± SEM. For all experiments, NKAES and FI-NK were assessed at day 3 or day 5 post-transduction, respectively.

We first transduced NK cells expanded using the Amplification and Expansion System (NKAES) and freshly isolated NK-cells (FI-NK) with an eGFP-encoding LV and observed that in both cases, BaEV-LVs outperformed VSV-G-, MV-, and RD114- LVs (Figure 1A, 83.4 % mean transduction rate vs. 15.7, 13.7, and 37.8% for NKAES, *p* < 0.0001, and 23.0% vs. 10.4%, 2.1 and 7.8% for FI-NK, *p* < 0.0001, respectively). The mean fluorescence intensity (MFI) of GFP after transduction in NK cells was similar for BAEV, VSV-G and RD114 and significantly lower for MV-LV in NKAES (Figure 1B). The mean transduction rate with BaEV-LVs was higher than 60% for NKAES even at low MOI of 1, and ranged from 12.4% at a MOI of 1 to a maximum of 27.2% at a MOI of 10 for FI-NK (Figure 1C). Transgene expression persisted over time after transduction with BaEV-LVs, although a decrease was observed from 70.6 to 61.4% in 14 days for NKAES (*p* = 0.06). Transduced FI-NK could be easily amplified after transduction (not shown). High transduction rates were also observed after NK-cell expansion on K562-mbIL15-41BBL feeder cells (8) or feeder-free NK MACS medium (15) (Figure 1D).

NK-cell receptors expression was assessed on untouched and BaEV-LV treated NKAES, which were either transduced (GFP+) or non-transduced (GFP-) (Figure 1E). There was no difference in CD56, CD16, NKG2D, NKG2A, NKp30, NKp44, and NKp46 receptors expression, suggesting that those markers are neither linked to the transduction efficiency, nor affected by the transduction (Figure 1E), unlike what has been recently reported (7). This difference could be attributed to the different expansion system used in our study.

**Figure 2:**
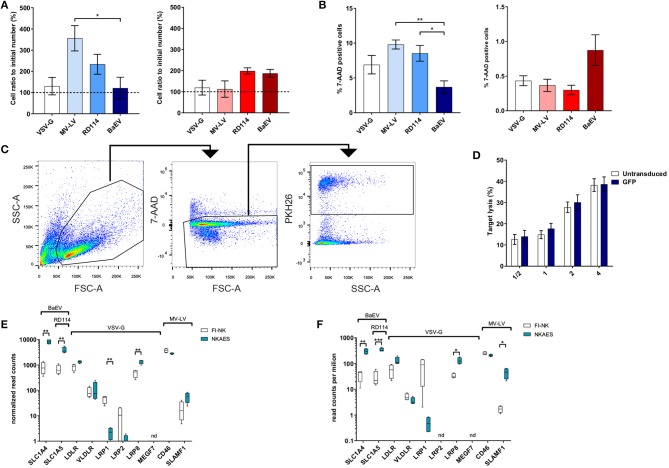
BaEV-LV transduction does not alter the phenotype or function of NK cells. **(A)** Viability of NKAES (left panel; *n* = 3) or FI-NK (right panel; VSV-G and MV-LV *n* = 6, RD114 *n* = 3, BaEV *n* = 9) with different LVs was assessed via the ratio of living (7-AAD-negative) cells in the culture normalized to the initial number of cells before transduction (*p < 0.05 and *p* = 0.141 for NKAES and FI-NK, respectively; one-way ANOVA test with multiple testing and Bonferroni correction). **(B)** Cell death, assessed by the percentage of 7-AAD positive cells, of NKAES (left panel; VSV-G, MV-LV and RD114 *n* = 3, BaEV *n* = 8) or FI-NK (right panel; VSV-G and RD114 *n* = 6, MV-LV *n* = 3, BaEV *n* = 12) with different LVs. Percentage of 7-AAD positive cells was calculated by flow cytometry after debris exclusion (*p < 0.05 and **p < 0.01 for NKAES, and *p* = 0.14 for FI-NK; one-way ANOVA test with multiple testing and Bonferroni correction). **(C)** Flow cytometry plot showing the gating strategy used in cytotoxicity experiments. Cells were first gated on forward and side scatter (FSC-A, SSC-A) and then the live 7-AAD negative cells were selected. Finally, PKH26 stained target cells were gated to numerate the remaining cells. **(D)** Cytolytic function of BaEV-LV-transduced vs. untransduced NKAES cells against K562 target cells. Data represent the percentage of target lysis, assessed by flow cytometry relative to the effector/target ratio and normalized to targets alone. (*p* = 0.4012, 2-way ANOVA test with multiple testing and Bonferroni correction, *n* = 3). **(E)** Quantitative expression of the viral receptors mRNAs assessed by RNA-seq in NKAES expanded with K562mbIL21 and in FI-NK-cells (*n* = 4, **p < 0.01, multiple *T*-tests with Holm-Sidak correction). **(F)** Quantitative expression of the viral receptors mRNAs assessed by RNA-seq in NKAES expanded with K562mbIL15 and in FI-NK-cells (*n* = 4, *p < 0.05, **p < 0.01, ***p < 0.001, multiple *T*-tests with Holm-Sidak correction). Data are presented as the mean ± SEM, except where noted. For all experiments, NKAES and FI-NK were assessed at day 3 or day 5 post-transduction, respectively.

The number of recovered living cells in both NKAES and FI-NK was preserved after transduction with BaEV-LVs (Figure 2A) although MV-LV transduction on NKAES yielded more living recovered cells than BaEV-LV transduction (*p* < 0.05). Although the percentage of dead cells in culture was low for all conditions (Figure 2B), it was higher in NKAES transduced with MV-LV and RD114-LV as compared to BaEV (*p* < 0.01 and *p* < 0.05, respectively). Together these results suggest that BaEV-LV transduction did not affect viability nor NK-cell proliferation.

We then assessed whether NK-cell cytotoxic function was preserved after BaEV-LV transduction and confirmed that the cytotoxicity of eGFP-transduced NKAES cells against K562 cells was equivalent to non-transduced NKAES (Figures 2C,D).

RNAseq analyses of both FI-NK and NKAES showed that ASCT1 and ASCT2 mRNAs were detected at significantly higher frequency in both IL-15- and IL-21-NKAES than in FI-NK (Figures 2E,F), which may explain the higher transduction rate of NKAES. These data were confirmed by qPCR (not shown). Also, the expression of both BaEV receptors by NK cells may explain the higher transduction efficacy of BaEV-LVs as compared to RD114 which use only one of those receptors.

**Figure 3:**
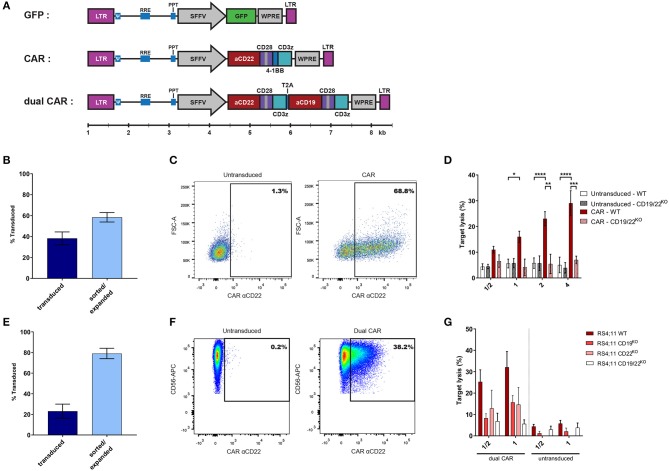
BaEV-LVs allow robust CAR-expression in NK-cells. **(A)** Schematic representation of the different LVs used for NK-cell transduction using BaEV-LV (relative scale according to size in base pairs). **(B)** Percentage of transduced NKAES cells using BaEV-LVs coding for an anti-CD22 CAR, assessed at day 3 after transduction (“transduced”; *n* = 15) and after sorting and 1 week of re-expansion (“sorted/expanded”; *n* = 3). **(C)** Flow cytometry plot representative of CAR-CD22 expression after NK-cell transduction with BaEV-LVs. **(D)** Cytotoxic assays of NKAES (either untransduced or CAR-CD22-NK-cells) against either parental (WT; *n* = 7) or CD19/22^KO^-RS4;11 cells (B-ALL; *n* = 2) (*p < 0.05, **p < 0.01, ***p < 0.001, ****p < 0.0001; 2-way ANOVA test with multiple testing and Bonferroni correction). **(E)** Percentage of transduced NKAES cells using BaEV-LVs coding for the dual CAR assessed at day 3 after transduction (“transduced”; *n* = 9) and after sorting and 2 weeks of re-expansion (“sorted/expanded”; *n* = 3). **(F)** Flow cytometry plot representative of dual CAR expression after NK-cell transduction with BaEV-LVs. **(G)** Cytotoxic assays using NKAES cells transduced with a dual CAR (left panel) or untransduced NKAES (right panel) against either parental (WT; *n* = 8 each), CD19^KO^ (*n* = 3 for untransduced, *n* = 4 for CAR transduced), CD22^KO^ (*n* = 1 for untransduced, *n* = 2 for CAR transduced) or CD19/CD22^KO^ RS4;11 B-ALL cells (*n* = 3 for untransduced, *n* = 5 for CAR transduced). Data are presented as the mean ± SEM.

CAR-expressing NK-cells represent one of the most relevant clinical applications of efficient NK-cell transduction. We first tested a single 3rd generation CAR construct recognizing CD22 (Figure 3A). We obtained a transduction rate of 38.3% ± 23.8% (mean ± SD) of NKAES and a high sustained level of CAR-expression (58.4% ± 7.8%; mean ± SD) after sorting and re-expansion (Figures 3B,C). We demonstrated that CD22-CAR-NK-cells efficiently and specifically killed B-ALL RS4;11 target cells, which were resistant to untransduced NKAES (Figure 3D). We could obtain 5 × 10^8^ CAR-expressing cells from 5 × 10^5^ transduced cells after an expansion of 14 days (not shown). Since transgene size affects transduction efficacy (16, 17), we also tested a dual CAR-expressing vector with two independent chains recognizing CD19 and CD22. The length of the dual CAR-CD22/19 LV had a significant impact on virus production and NK transduction was lower (23.1 ± 20.5%; mean ± SD) (Figures 3E,F). However, we were able to sort and re-expand these dual-CAR-transduced NK-cells for 2 weeks, keeping the transgene expression at a high level (79.0 ± 8.7%; mean ± SD) (Figure 3E). These CAR-CD22/19-NKAES killed efficiently CD19^KO^ or CD22^KO^-RS4;11 cells, which suggest that this strategy could be efficient for preventing tumor evasion to CAR therapy (18, 19) (Figure 3G).

In this study we showed that BaEV-LV is an efficient and robust tool to transduce NK cells. As a proof-of-concept, we generated large numbers of engineered CAR-NK-cells, which induced specific killing of antigen-bearing cancer cells, even with a large dual CAR-LV construct. This technique was robust and reproducible in different expansion systems, including a feeder-cell-free system. The higher level of transduction could open up possibilities for the use of this method to generate an immunotherapeutic product. The prevalence of receptors, as seen by RNAseq, could explain the difference seen between the transduction of activated and resting NK cells. The fact that activated NK cells express both entry receptors at high level could also explain why this envelope protein is more efficient than the others. The development of such a tool could have a major impact on both basic research of NK-cell biology study and NK-cell-based immunotherapy.

## Data Availability Statement

The datasets generated for this study have been deposited in GEO database of NCBI (#GSE128696, #GSE129044).

## Ethics Statement

The studies involving human participants were reviewed and approved by the institutional ethical board of the CHU Sainte-Justine (approved protocol #CER-3527). The participants (healthy volunteers) provided their written informed consent to participate in this study.

## Author Contributions

AC, WL, PB, SN, HR, SS, MG, and CT-L performed the experiments. AC, WL, PB, and KB wrote the manuscript. NC and DL generated the RNAseq data on IL-21 expanded NKAES cells and participated in the redaction of the manuscript. JS and LB generated the RNAseq data on IL-15 expanded NKAES cells and participated in the redaction of the manuscript. RD recruited participants and collected samples. EH generated the hypotheses, conceptualized the study, and wrote the manuscript. EV provided BaEVTRless encoding plasmid, discussed results, and wrote the manuscript. All authors reviewed and approved the manuscript.

### Conflict of Interest

EV has a patent EP2761010 licensed to Lentigen/Miltenyi Inc. The remaining authors declare that the research was conducted in the absence of any commercial or financial relationships that could be construed as a potential conflict of interest.
